# Energy saving analysis for pump-motor set in water purification plant using variable speed drive

**DOI:** 10.1038/s41598-024-75601-z

**Published:** 2024-11-12

**Authors:** Mohamed Adel Esmaeel Salama, Nada Mohamed El-Naggar, Salama Abu-Zaid

**Affiliations:** 1 Department of Electrical Power and Machines Engineering, Faculty of Helwan Engineering, Helwan University, Helwan, Egypt; 2 Department of Electrical Power and Machines Engineering, Faculty of Engineering, Al-Azhar University, Cairo, Egypt

**Keywords:** Variable speed drive, Induction motor, Energy consumption, Pumped-storage power stations, Pumping plants, Electrical and electronic engineering, Energy grids and networks, Power stations

## Abstract

The induction motor is known to be the most reliable motor in the industry and is also the most energy-consuming load worldwide. It is noticeable in some production areas that the use of a high-capacity induction motor is not required at some operating times, and therefore it is important to control the speed of the induction motor to suit the operating requirements and obtain highly efficient performance. This paper presents a case study of a drinking water purification plant that uses a variable speed drive (VSD) as a speed controller for one of its induction motors to drive the pump. The VSD is connected to the motor in question to regulate its speed and maintain the external pressure of the station at an appropriate value that ensures water delivery to all branches of the network. 24-hour power consumption measurements of the respective engine with and without the use of a VSD are studied and analyzed to determine the potential for energy savings. Finally, a case study simulation is presented to compare the simulation results with the actual results.

## Introduction

Due to the increased demand for electrical energy consumption and the need for large amounts of fuel to generate electricity, it is important to reduce the use of electrical energy while obtaining the same results required for the user. Many authors have published articles on saving electrical energy at various industrial sites. Reference^1^ case study was carried out at the St. Combs Tea Factory, which monitors the electrical power consumption of two identical 4 kW motors. One of the motors is controlled by a conventional inlet damper method, while the second is controlled by a VSD, and from the study, there was an energy saving of 40%. Reference^2^ In Saudi Arabia, 60% of the energy consumed in homes is wasted on air conditioning systems. A comparison of the energy performance of two identical 5-ton rooftop air conditioning systems installed in two identical homes was performed. The first air conditioner works as a conventional on/off strategy, and the second is controlled by a VSD. When the room temperature is high, the VSD increases the motor speed to provide greater room cooling until the desired temperature is reached, and vice versa. As a result of the comparison, energy savings range from 22 to 65% in March, which means that VSD achieves promising results in the field of energy savings. Reference^3^ The sugar factory has two steam boilers to produce steam with a flow rate of 65 tons per hour at a temperature of 400 °C and 30 bar. An induced draft (ID) fan with a rated power of 400 HP is used in the boiler to remove air and flue gases and ensure proper negative pressure in the combustion chamber. The ID fan rotates at a constant speed at minimum and maximum boiler operation. In order to ensure accurate and constant steam supply to the plant with efficient energy consumption, it is proposed to install a VSD in the ID fan motor to control the motor speed and then control the air or gas flow (pressure). A study was conducted on the ID fan power consumption before and after using VSD to calculate the energy savings; the energy saving percentage was 47% .

Among the previous cases, the problem is that the traditional control method, such as controlling the output of the pumps, increases the loads on the motor and thus increases the energy consumption that is not required for the user. The second traditional method is to disconnect and connect electric motors. The problem with this method is that repeatedly disconnecting and connecting leads to a high starting current passing through the number of times disconnecting and connecting.

**Fig. 1:**
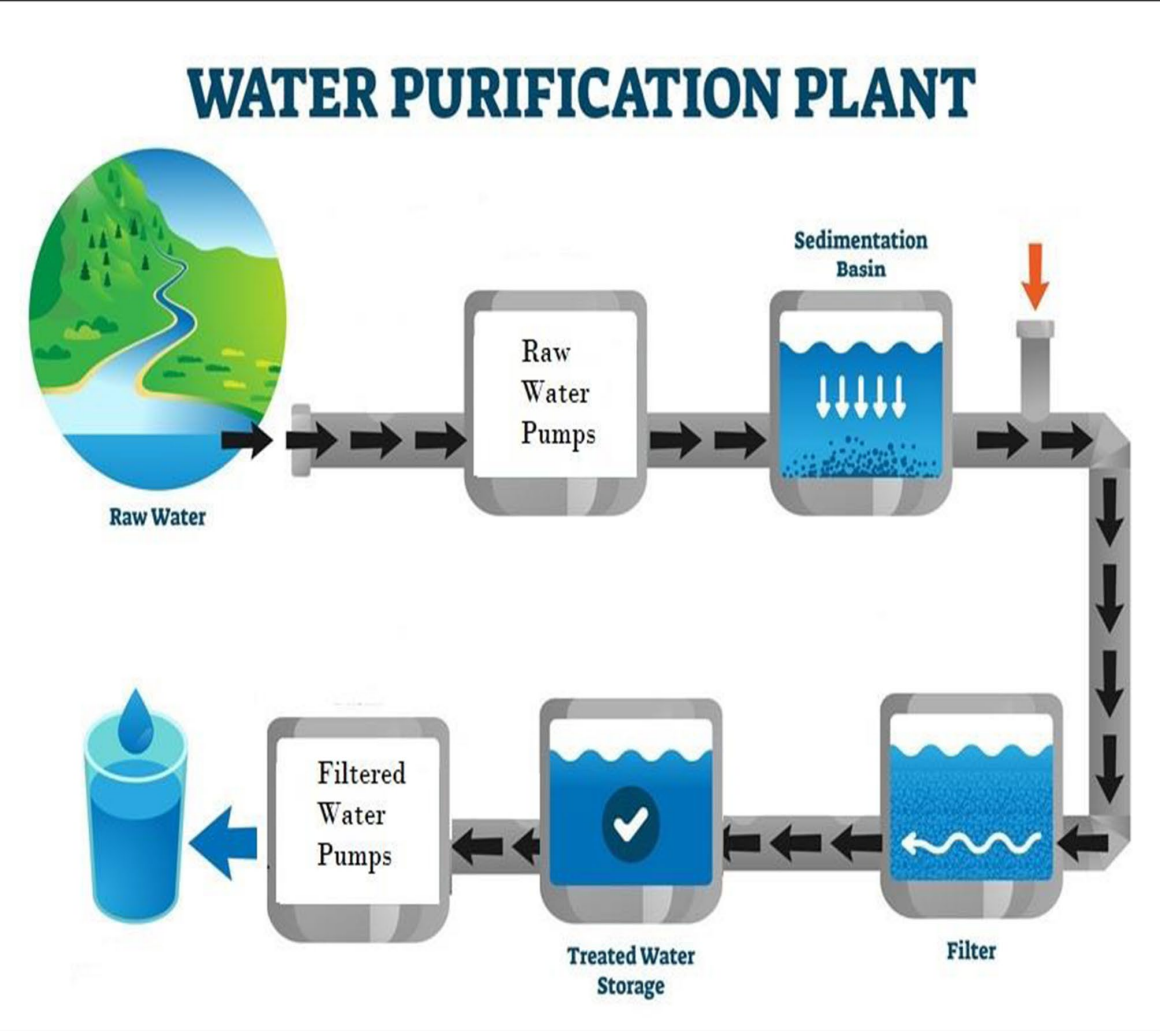
Scheme of the direct filtration water purification plant.

In this research, an energy-saving study was conducted for a drinking water purification plant in a village in southern Egypt. The station contains two pumps, one of which is controlled once by the traditional method, which is the throttle on the flow valve, and the other time the same pump is controlled using VSD technology, and the results are compared. The study was conducted using Matlab simulation programs as well as site results from inside the drinking water station, which has a population of about 30,000 people. The plant is designed to have a full capacity of 200 L per second using direct filtration purification technology, as shown in Fig. 1, Refs.^4–6^.

- Intake and the raw water ward: The raw water pumps take the raw water from the river into the plant to begin the purification process. Some chemicals like aluminum sulfate (Al₂(SO₄)) and chlorine are added to the water before entering the filters.
- Filters: The filters consist of several layers of filtration media like sand to purify and remove the plankton from the water. Then the filtered water is collected to the reservoir.
- Filtered water ward: The filtered water pumps take the water from the reservoir to supply the out network with high pressure water (4 bar for the case study plant), Ref.^7^.

**Fig. 2:**
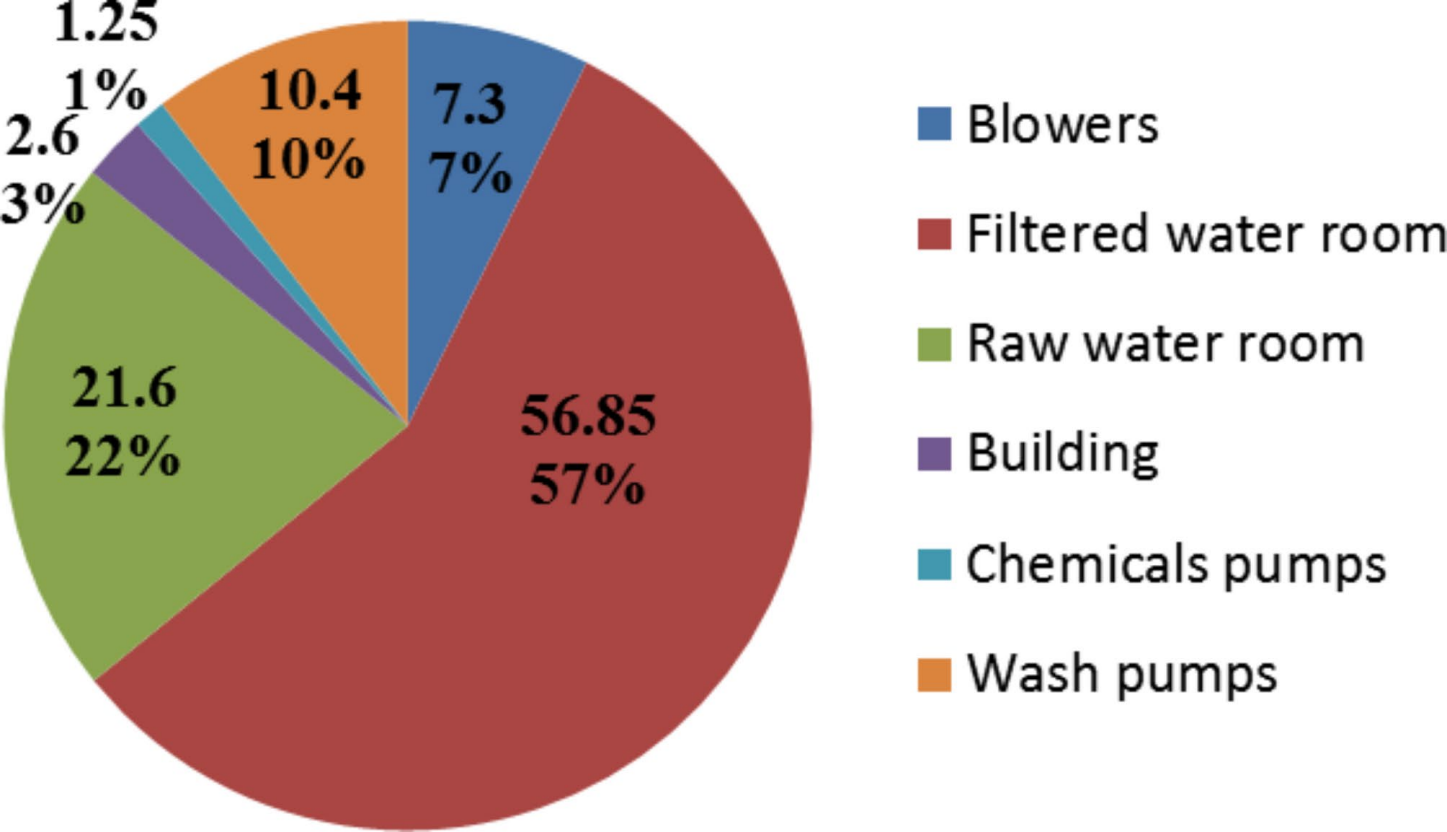
Disparity of the energy consumption for different plant wards.

The 200 L/s plant capacity is higher than the network demand at most of the day time. Though in case of operating the plant at the full capacity over the day, the out head of the plant rises over the desired value which poses threats to the network (pipes or valves destruction). Since to maintain the output head of the plant at an appropriate value (4 bar) to protect the network and also ensure delivering the water to the last terminal of the network, some inefficient control methods like throttling are done to the equipment (pumps-motors) especially in the filtered room which considered the most energy consumer in the plant as Fig. 2 shows.

### The operation of the filtered ward

**Fig. 3:**
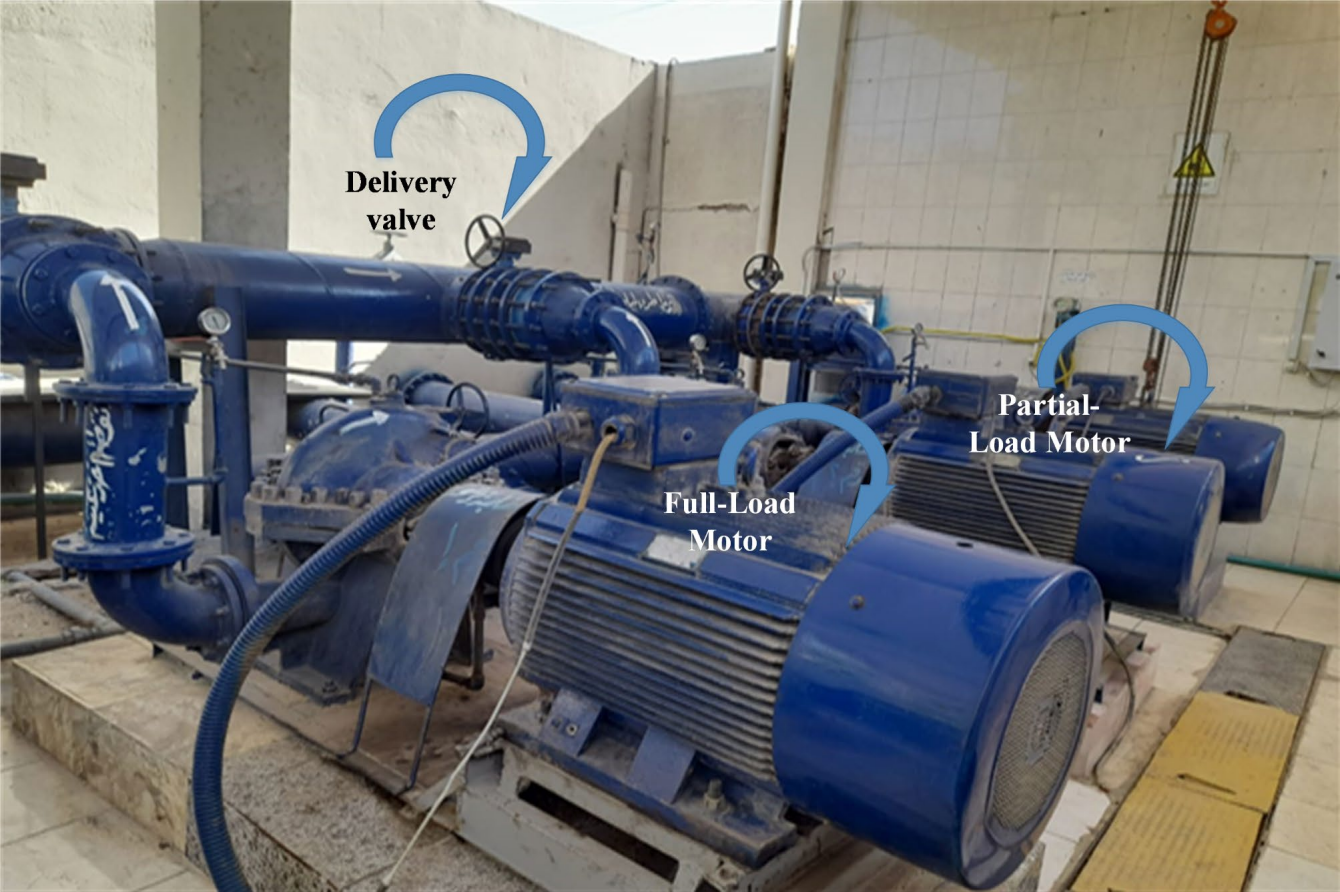
Filtered ward pumps and motors.

The filtered ward has a specific operation according to the equipment number and rating. The filtered ward is responsible for delivering the treated or filtered water from the reservoir to the network with high head (pressure). It is electrically supplied through a 630 kVA, 11 kV/0.4 kV transformer. The filtered ward has three identical pumps with 110 $\:\text{l}\text{i}\text{t}\text{r}\text{e}/\text{s}\text{e}\text{c}\text{o}\text{n}\text{d}$ flow and 60 m head pressure each as Fig. 3, these pumps are driven by three phase induction motors with data as Table 1 with rating 400 V, 132 kW, 4-poles, 50 Hz, 1490 rpm.

**Table 1:** Filtered ward motors data and pumps data.

Induction motor of centrifugal pump	Cos ϕ	Connection	Flow rate	Head or pressure
	0.88	Y/Δ	369 (m^3^/h)	60 m
Frequency	Rated power	Rated voltage	Rated current	Rated speed
50 Hz	132 kω	690/400 V	191.7/227 A	1490 rpm
60 Hz	158 kω	830/480	191.7/227	1788 rpm

### The normal operation of the filtered ward with throttling

The default operation of the filtered water ward is to fully turn on pump number 1 as a base load, and controlling pump number 2 flow’s by throttling the delivery valve according to the network demand through the day (Pump 3 is spare). Flow and pressure meter with data logger are fixed on the discharge line (pipe) of pump (2) to record the water flow required over the day. It is found that the ratio between the actual required flows to the rated flow of pump 2 that maintain the output pressure of the plant at 4 bar that guarantee water delivery to the network terminals all day long as Table 2.

**Table 2:** Load factors for pump (2) for 24 h.

Load factor	100%	90%	80%	70%	Lower 70%	
Day time ratio (24 h)	0	8.3%	54.2%	37.5%	0	
Number of hours/day	0	0.75	13	10.25	0	Total = 24 h

That means that pump (2) operates at partial load all of the day. So using throttling which is an inefficient method to control the flow would cause much loss in energy consumption, Ref.^8^.

### The operation of filtered ward with variable speed drive

**Fig. 4:**
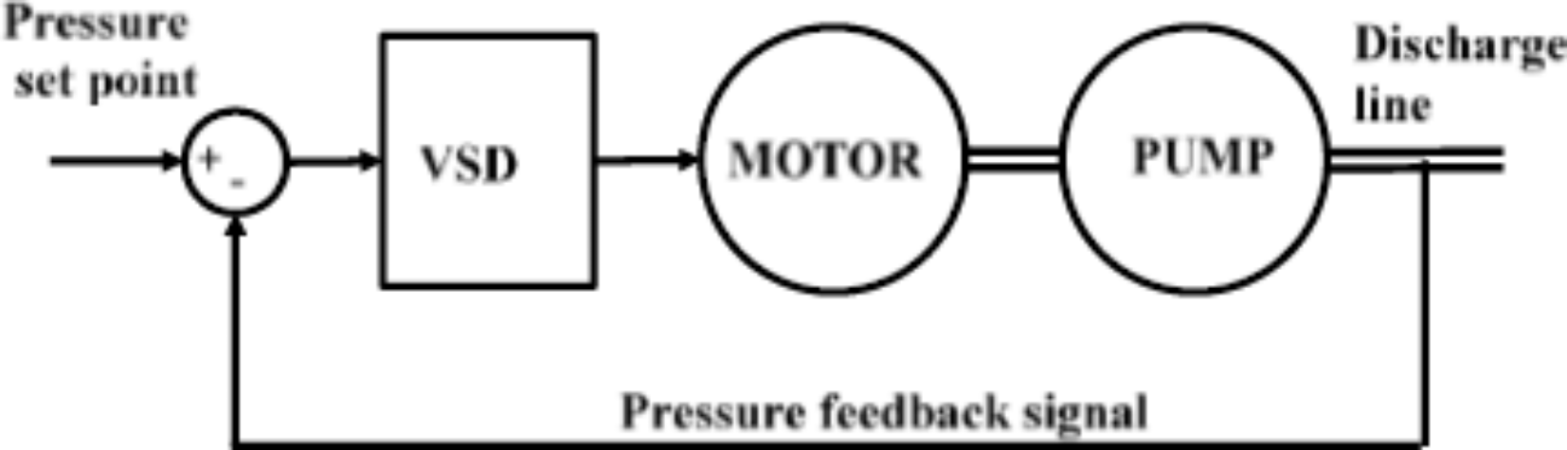
Closed loop control of the plant pressure using VSD.

Therefore, it is suggested to install VSD on motor 2 to be able to efficiently control the flow of pump 2 by varying the motor speed. The closed loop control scheme of the system is shown in Fig. 4. The pressure of the network delivery pipes is measured by pressure meter (it supposed to be 4 bars), the error between the reference or The VSD controls the voltage magnitude and frequency according to the control signal to change the speed of the motor rotor and thus the pump speed changes the pump flow to maintain the pressure at the desired value (note that the first pump operates at the rated load), Refs.^9,10^.

**Fig. 5:**
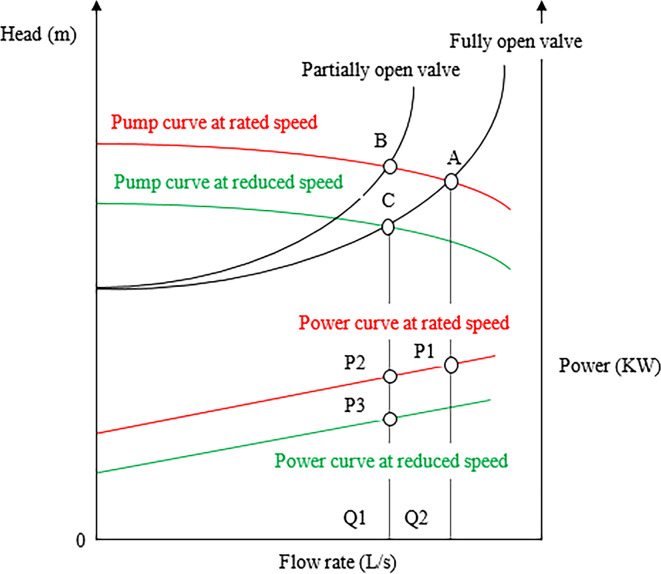
Centrifugal pump performance curves.

In addition to providing pressure control, a VSD can save a significant amount of energy consumption^6^. The characteristic curves of the pump with choke and VSD are as in Fig. 5.

Point A represent the operating point at full open valve (the intersection between the pump curve at rated speed and system curve at fully opened valve) which corresponds to power P1 at the power red curve. While point B is the operating point during throttling (the intersection between the pump curve at rated speed and system curve at partially opened valve) the flow is reduced and the power is slightly reduced at power P2.

Point C is the operating point during using VSD (the intersection between the pump curve at reduced speed and system curve at fully open valve) high drop in power at P3.

It is obvious from Fig. 5 that the power at reduced speed (P3) is lower than the power at throttling method (P2). So energy saving is achieved by VSD as a speed controller.

### The energy consumption calculations for the case study motor

The consumption of the energy before and after using VSD as well as the energy savings is measured by two different methods.

- The MATLAB/SIMULINK program.
- The actual measurements using the power analyser at the plant.

### MATLAB/SIMULINK model and calculations

The MATLAB/SIMULINK program is used to simulate the case study to audit the energy consumptions calculations by using throttling method as a flow controller and by using VSD as a speed controller for the afore mentioned motor (motor two).

### Using throttling as a flow controller

**Fig. 6:**
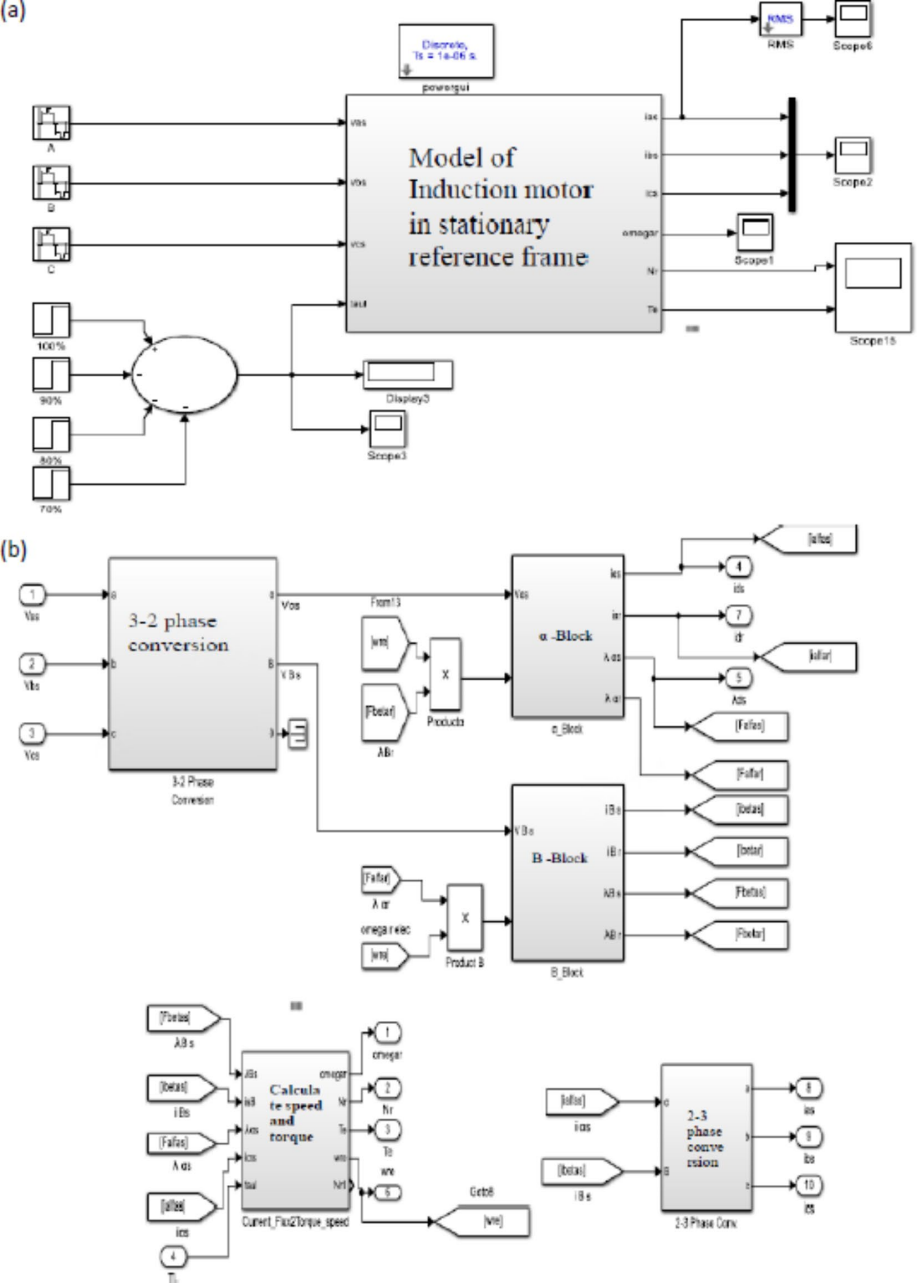
(**a**) Model of motor 2 with throttling. (**b**) IM model in stationary reference frame.

The model in Fig. 6a includes a three-phase voltage source with a line-to-line RMS value of 400 V connected to an induction motor model having a rated value and parameters are summarized in Table 3.

**Table 3:** Motor ‘two’ parameters referred to stator side and data.

Rated voltage	Rated power	No of pole	Frequency	Rated torque
400 V	132 kW	4	50	864 Nm
Stator resistance	Stator leakage inductance	Rotor resistance	Rotor leakage inductance	Mutual magnetization inductance
13 mΩ	152 mH	13 mΩ	152 mH	6.69 mH

The induction motor is represented using the Park transformation which change the three phase variables into a two phase variables in the Alfa-Beta stationary reference frame as shown in Fig. 6b and the model is given in Refs.^11,12^.

The motor is loaded with different values of loads 100%, 90%. 80% and 70% of the rated load in steps as Table 4.

**Table 4:** Simulation output readings for the motor using throttling.

Time (s)	% Loading	Irms (A)	P.F	⍵ (rad/s)	T_d_ (Nm)	Pin (kω)	Energy (kω h)
3–6	100	230	0.85	156.1	845	132	0
6–9	90	212.6	0.831	156.3	762.8	122.4	91.8
9–12	80	195	0.805	156.4	678.5	108.755	1414
12–15	70	178.2	0.77	156.5	593.9	95	974
							2479.8

The input power and energy of the motor can be determined using the following equation, Ref.^13^:

1 $$\:{\text{P}}_{\text{i}\text{n}}\left(\text{k}\text{W}\right)=({\text{s}\text{q}\text{r}\text{t}\left(3\right)\text{*}\text{V}}_{\text{r}\text{m}\text{s}}\left(\text{V}\right)\text{*}{I}_{rms\:}\left(Amp\right)*P.F)/1000$$

2 $$\:{\text{E}}_{\text{c}\text{o}\text{n}\text{s}\text{u}\text{m}\text{p}\text{t}\text{i}\text{o}\text{n}}\left(\text{k}\text{W}\text{h}\right)={\text{P}}_{\text{i}\text{n}}\left(\text{k}\text{W}\right)\text{*}{h}_{operating\:}\left(h\right)$$

**Fig. 7:**
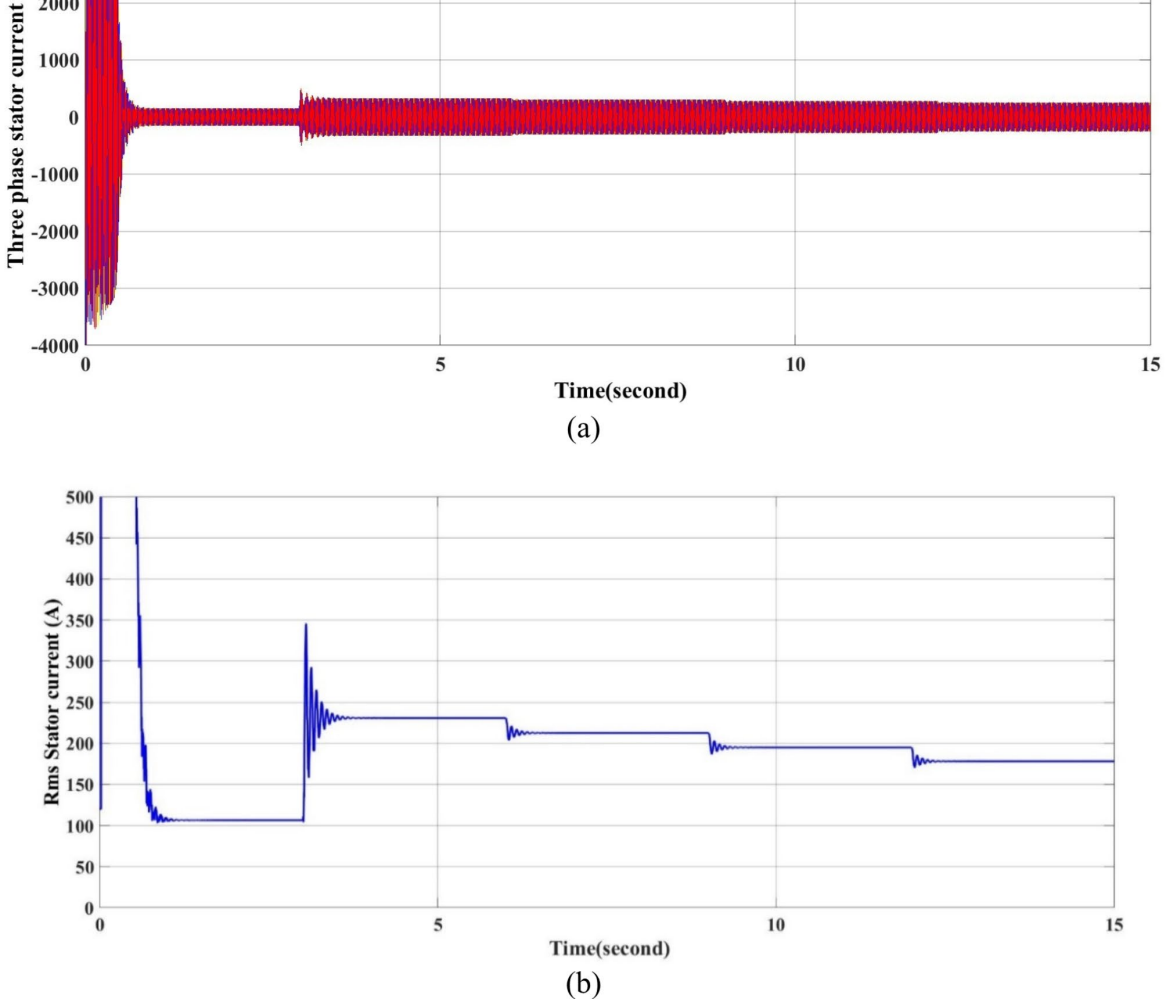
Current output waveforms of the model by using throttling. (**a**) The three phase motor stator current and (**b**) the RMS current for phase A.

The current output waves are shown in Fig. 7a, b. The three-phase and RMS current decrease as the load torque decreases at times 6, 9 and 12 s, as shown in Fig. 7a and b, respectively.

**Fig. 8:**
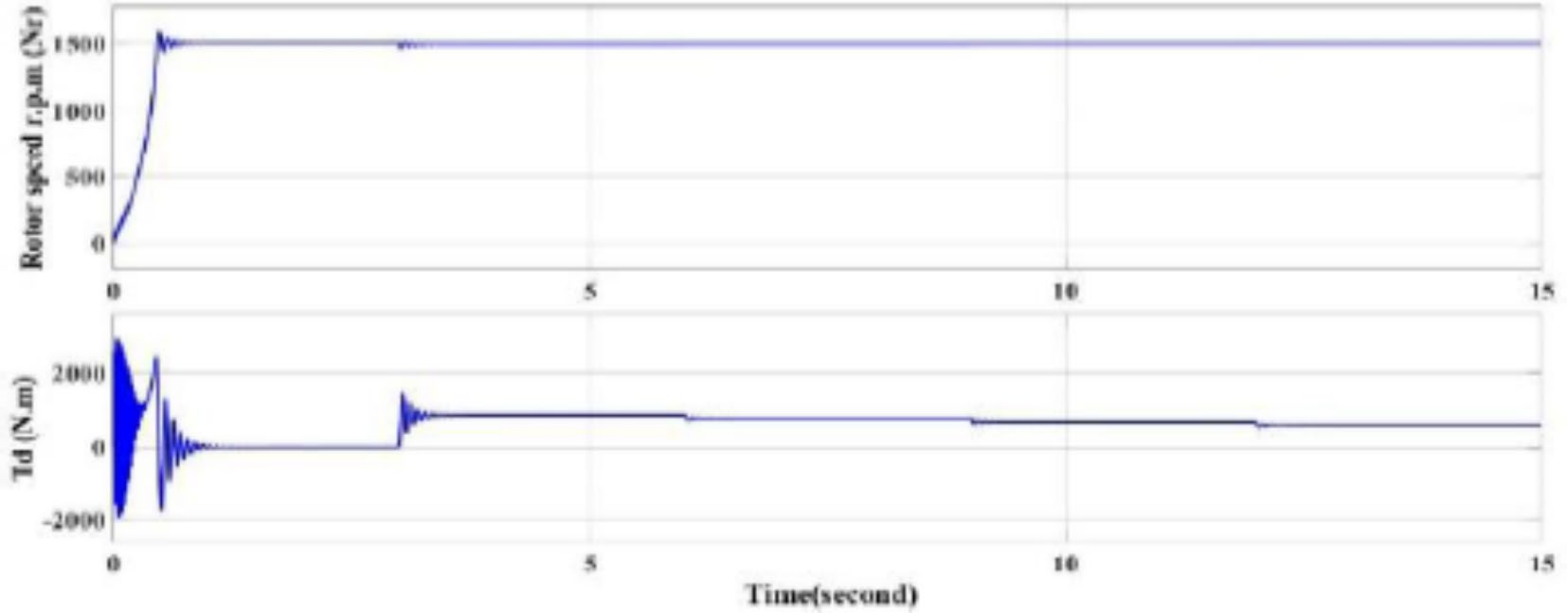
Rotor mechanical speed and the developed torque of the motor by using throttling.

While the rotor speed is slightly increasing by 1 or 2 rpm (almost constant) by decreasing the load torque and the developed torque slightly reduces as Fig. 8.

### Using VSD as a speed (flow) controller

**Fig. 9:**
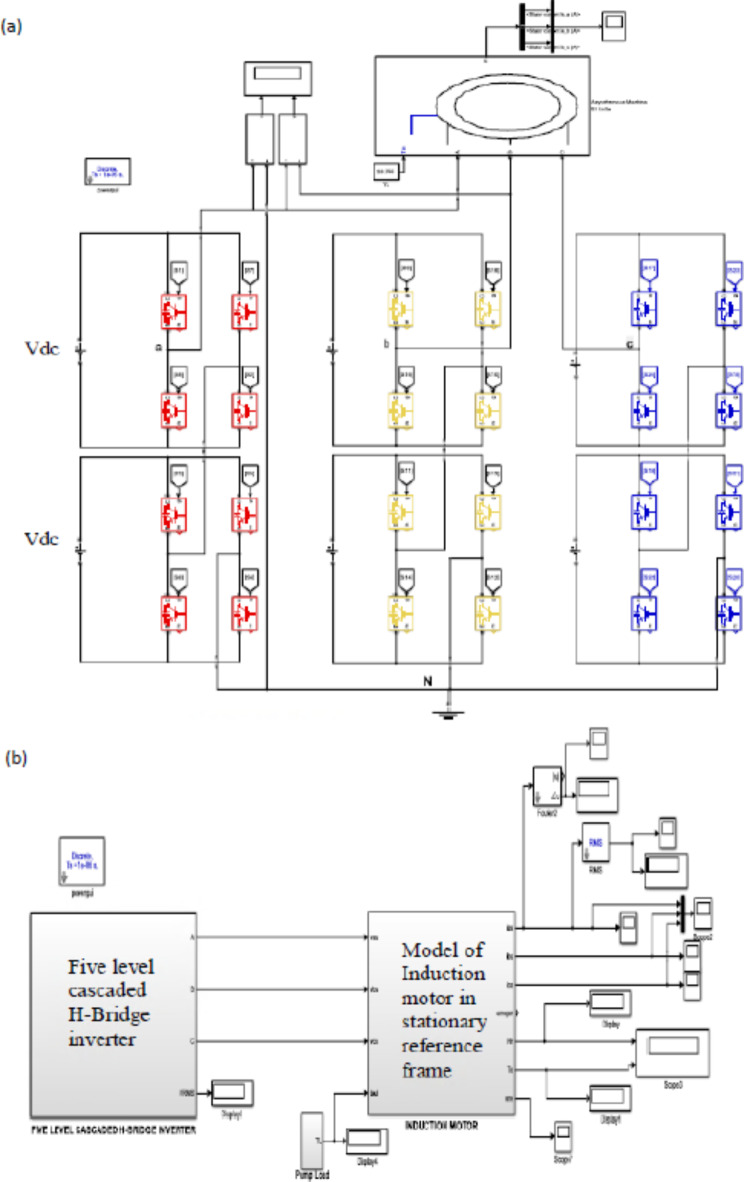
(**a**) Model of the five-level CHB inverter. (**b**) Model of motor 2 with VSD.

The five-level cascade H-bridge inverter model, as shown in Fig. 9a, consists of a (three-phase) topology supplied by two isolated DC sources with VDC per phase. This model is available as a Refs.^11,12^.

The inverter is connected to an induction motor with parameters as shown in Table 3 and loaded with a pump as shown in Fig. 9b. This inverter is fed with a set of signals coming from a control unit called a PI controller. The PI controls the inverter and thus controls the induction motor of the pump, so that the following Eq. (3) is achieved, Ref.^13^

3 $$\:{T}_{max}\:=\frac{P}{4\pi\:}\:\:{\left(\frac{{V}_{1}}{{f}_{1}}\right)}^{2}\frac{1}{4\:\pi\:\:{L}_{eq}^{{\prime\:}}}$$

Where:

T_max_: Maximum torque

P: No of pole.

V_t_: Terminal voltage.

F_1_ : Supply frequency.

L_eq_: : The equivalent Inductance of the motor windings.

From time (0 to 3) second the motor is softly start from zero to rated speed, at time 3 s the motor rotates at the 100% of the rated speed then at time 6 s the motor rotates at 90% of the rated speed, while at time 9 s the motor rotates at 80% of the rated speed. Finally, at time 12 s the motor rotates at 70% of its rated speed (the rated speed is 1490 r.p.m). The reference speed is simulated and the simulation results as The input power and energy of the motor can be determined using Eqs. (1) and (2) and noted in the Table 5 for different loading cases. The pump load simulated by taking the rotor feedback speed and calculated the corresponding torque as the torque is directly proportional to the square of the speed in rotary loads (pumps).

**Table 5:** Simulation output readings for the motor using VSD.

Time (s)	% Loading	V_rms_ (V)	$\:{\text{I}}_{\text{rms}}$ (A)	F (Hz)	P.F	⍵ (rad/s)	T_d_ (Nm)	Pin (kω)	Energy (kω h)
3–6	100	401.9	230.9	49.92	0.848	156	852	132	0
6–9	90	361.7	196.6	44.9	0.828	140.4	699	101.9	76.5
9–12	80	323.9	168	39.89	0.783	124.8	554	73.7	959.4
12–15	70	285	145.4	34.89	0.739	109.2	426.3	53.4	547.35
									1582.25

**Fig. 10:**
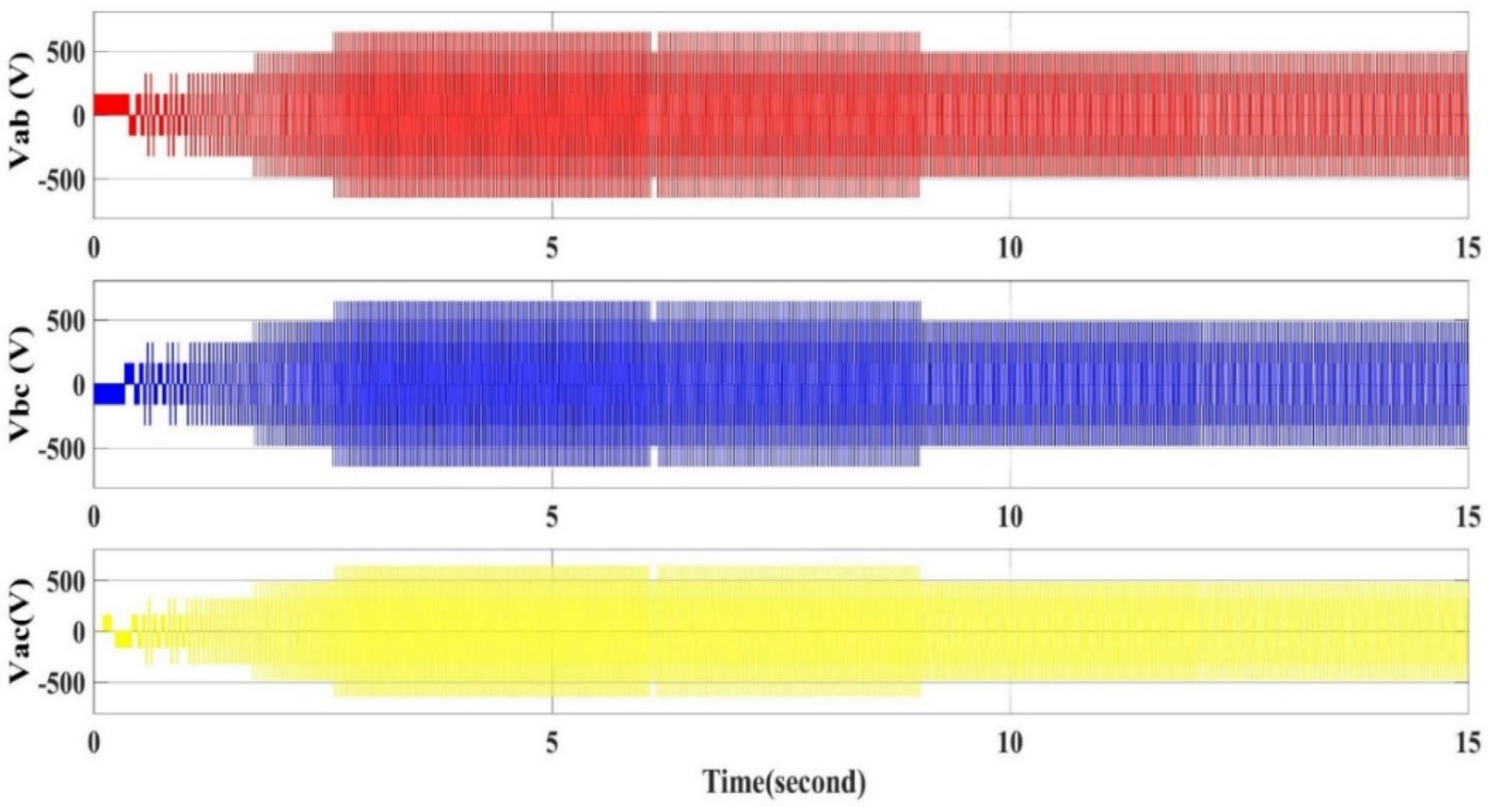
Inverter output three phase line voltages for all conditions of reference speed.

Based on the control PI the output voltage from the inverter is changed, as shown in Fig. 10. The three-phase line voltage of the inverter is varied when trying to control the motor speed using the V/F control method.

**Fig. 11:**
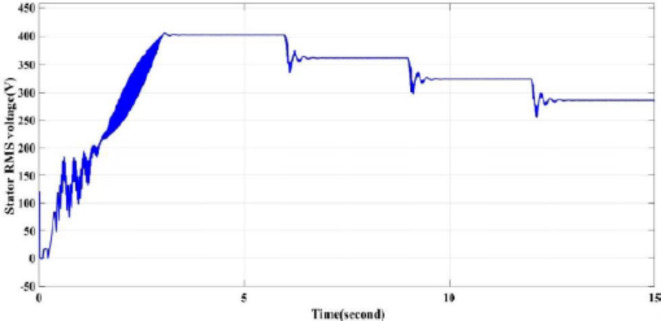
Inverter output line voltage in RMS for all reference speed conditions.

**Fig. 12:**
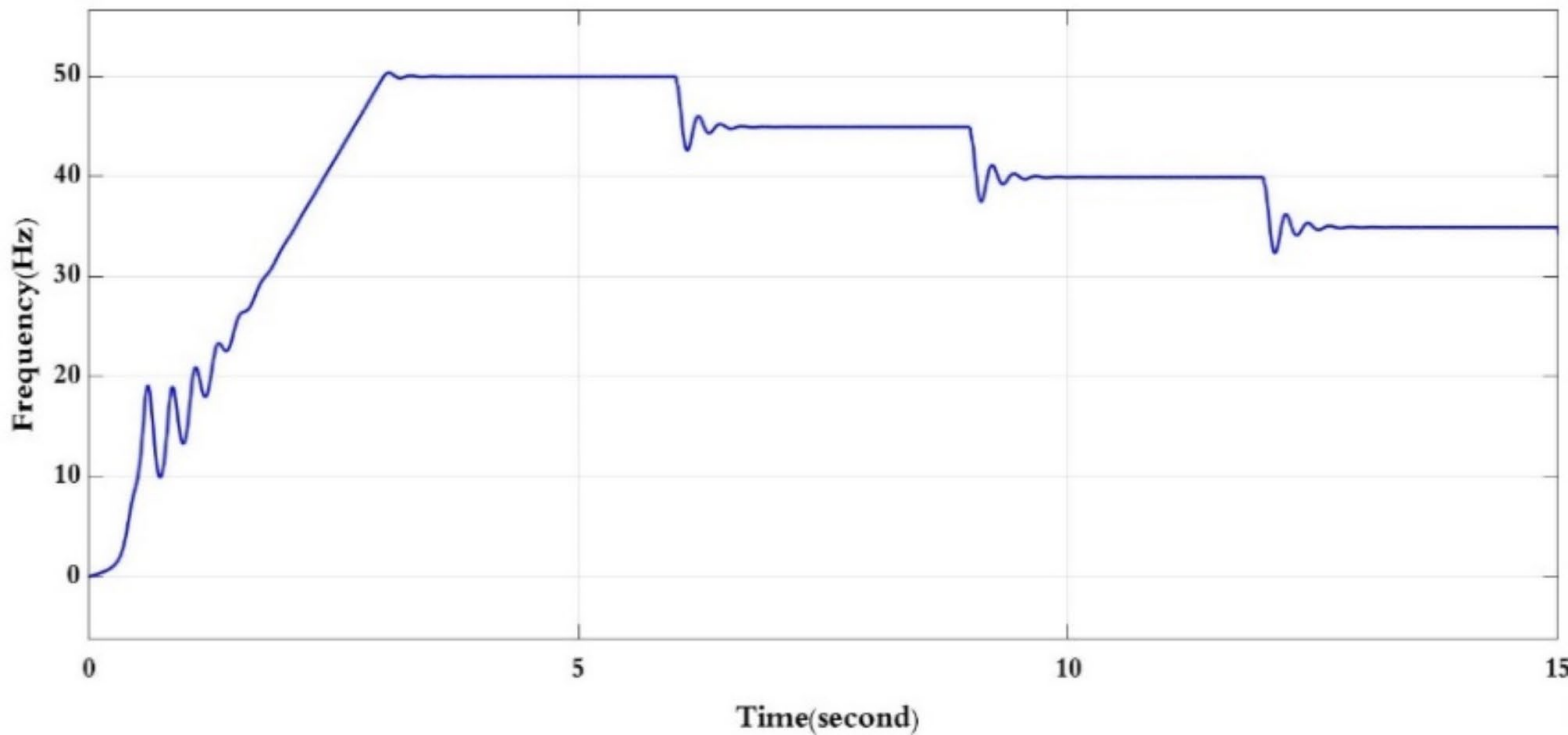
Inverter output frequency for all reference speed conditions.

Figures 11 and 12 the RMS output voltage and frequency as a result of changing the reference desired speed.

The RMS voltage and frequency increase at starting till reach the rated to time 3 s, from second 3 till reach 6 s the voltage and frequency are at rated value as the reference speed is at rated speed, then from second 6 to 9 the voltage and frequency are reduced to match the reduction in the reference speed (90% of the rated speed), then from second 9 to second 12 the voltage and frequency reduced again as the reference speed is reduced to 80% of the rated speed. Finally, from second 12 to 15 the voltage and frequency are reduced another time as Figs. 11 and 12.

**Fig. 13:**
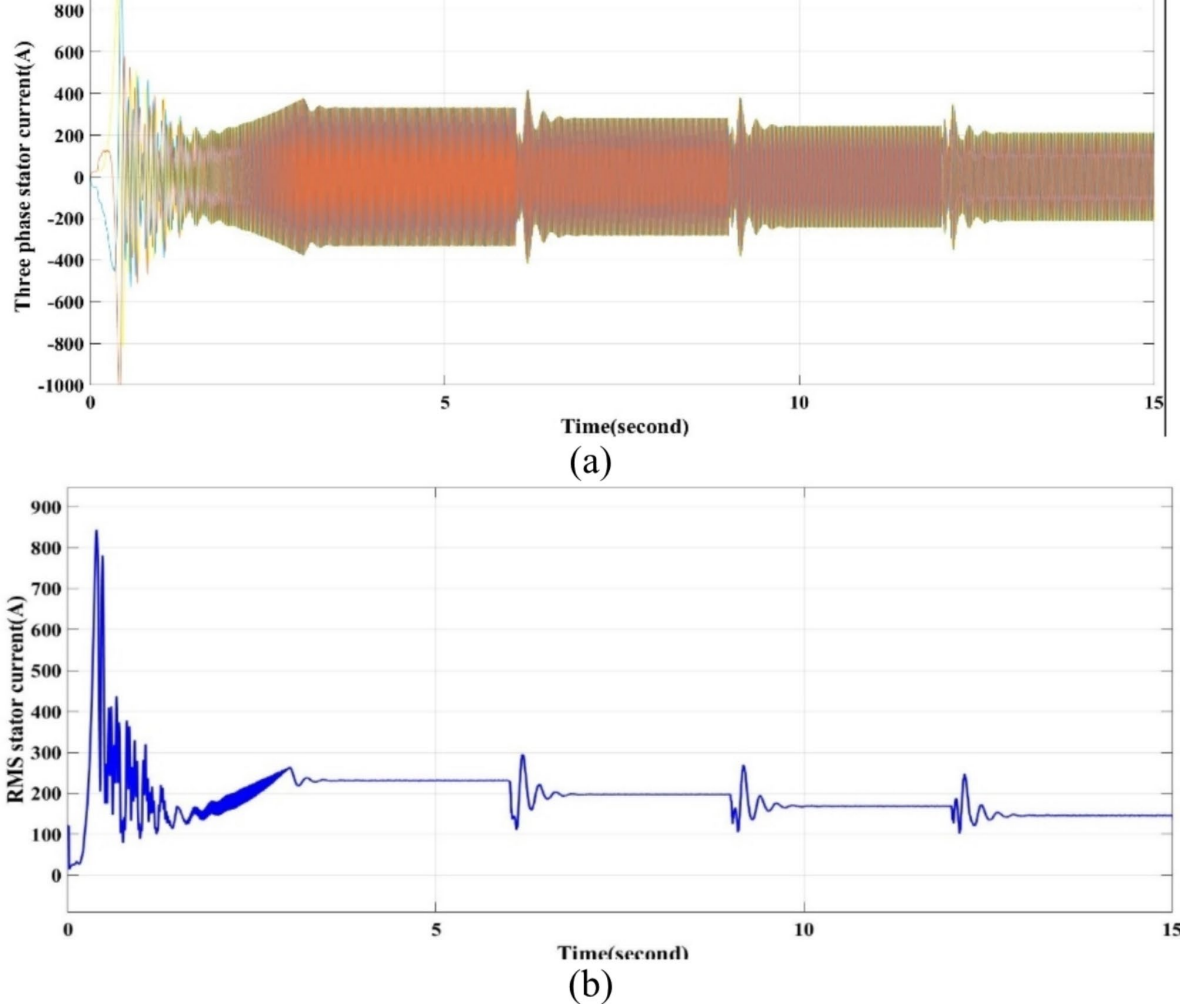
Motor stator current for all conditions of reference speed with VSD. (**a**) The three phase motor stator current and (**b**) the RMS current for phase a.

The stator three phase current and RMS current for phase ‘a’ are begin starting till time 3 s, then reach the rated from 3 to 6 s. Then begin decreasing step by step with reducing the reference desired speed at time 6, 9 and 12 s as Fig. 13a, b.

**Fig. 14:**
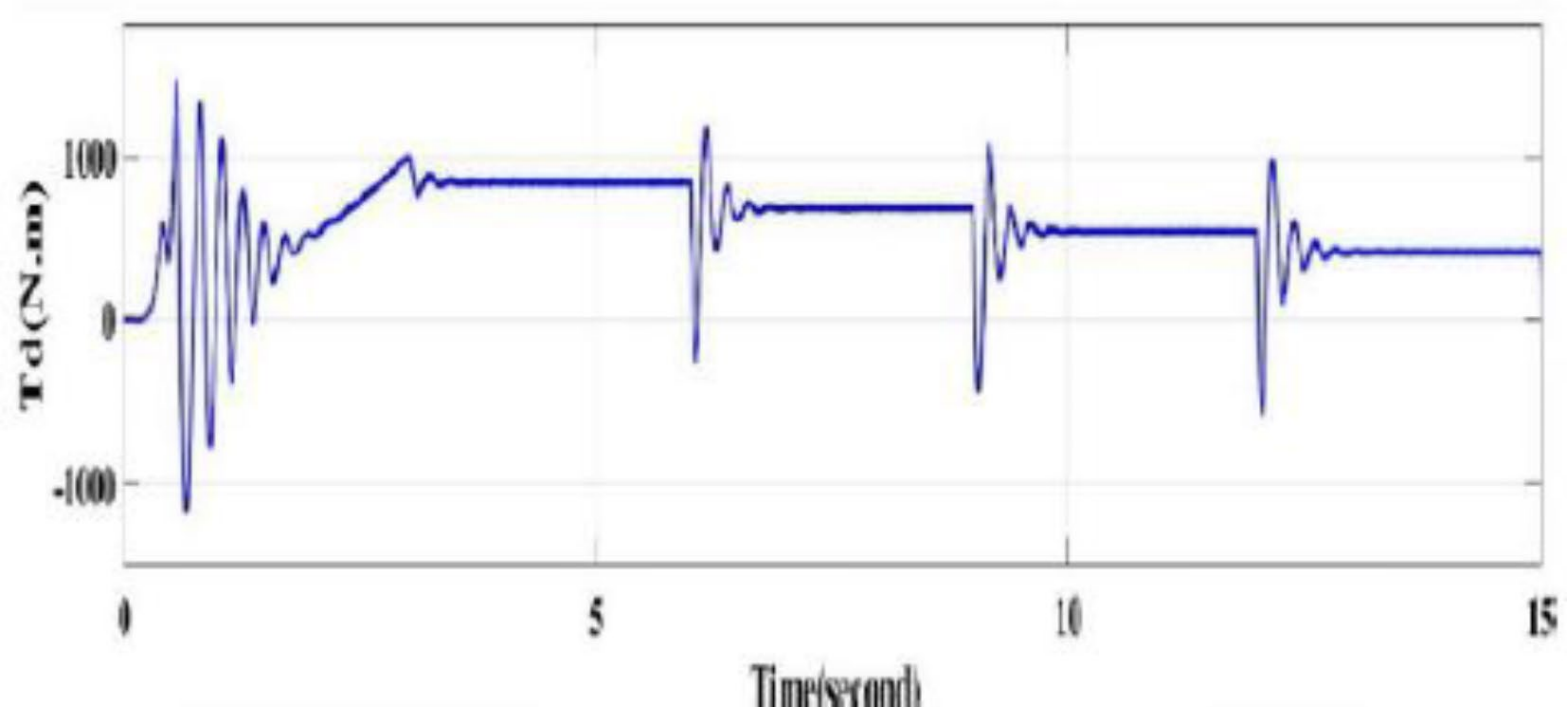
Developed torque with VSD.

**Fig. 15:**
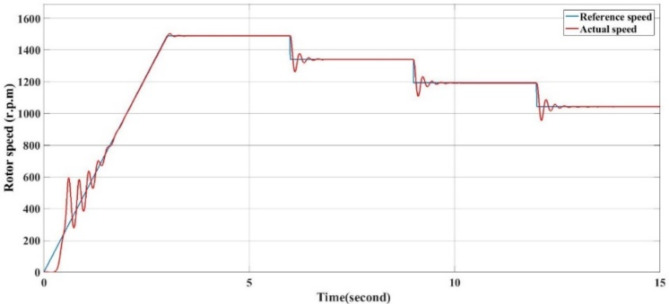
Reference speed vs. actual speed with VSD.

The rotor actual mechanical speed increasing from zero till gets the rated speed then trying to match the reference desired speed at all conditions. The torque oscillates at starting till reach the rated torque decreases as a result of the reference speed reduction as Figs. 14 and 15.

### Energy saving calculations upon simulation results

**Fig. 16:**
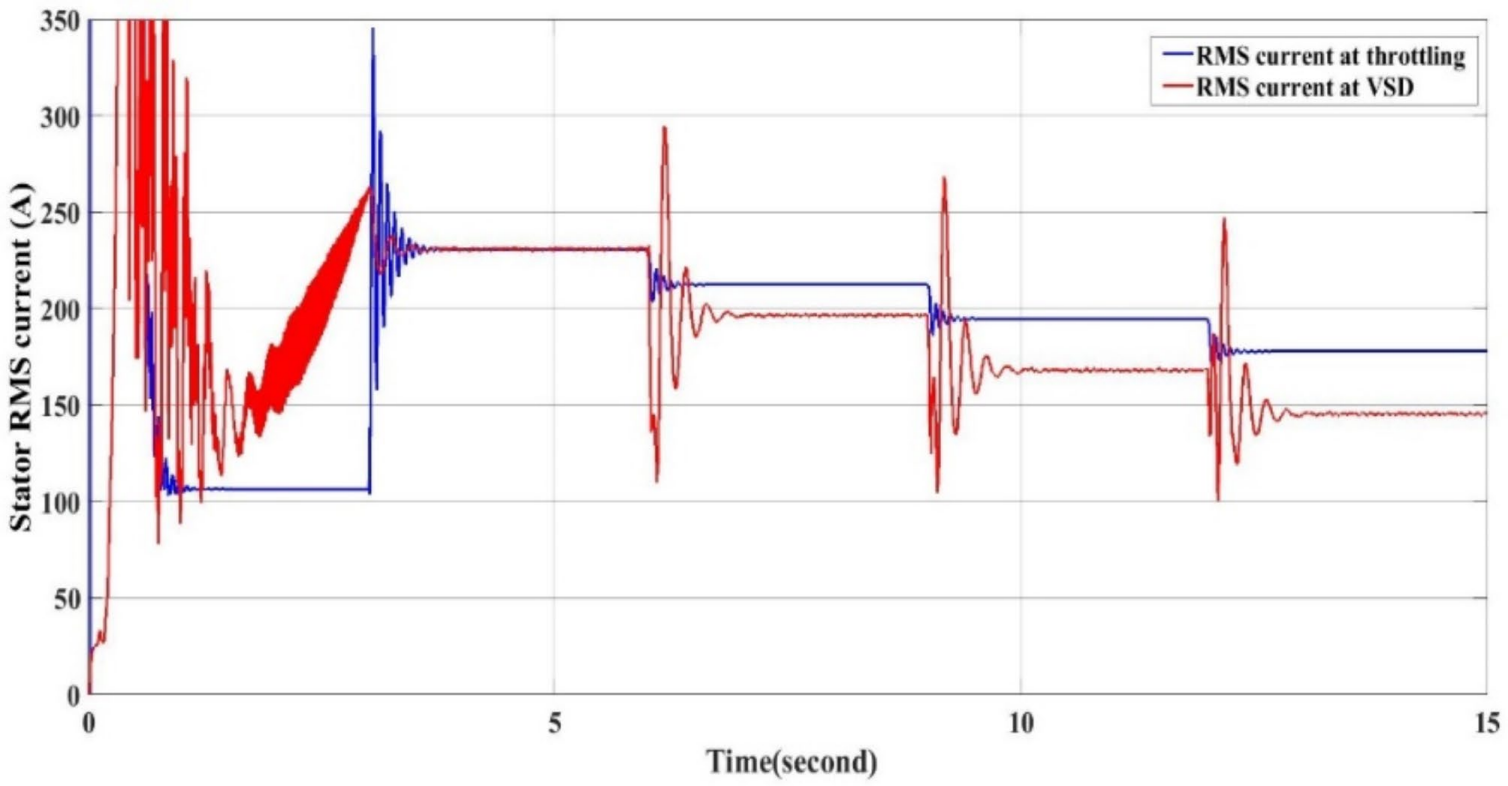
RMS current with throttling and VSD.

The energy saving due to the reduction in the current demand during using VSD as Fig. 16 is calculated in the following equations.

Using the results in Tables 4 and 5 the value of daily energy consumption using throttling is 2.48 MWh and the value of daily energy consumption using VSD is 1.579 MWh and Energy saving is Ref.^13^

4 $$\:{\text{E}}_{\text{s}\text{a}\text{v}\text{i}\text{n}\text{g}}=\:2.48\:\text{M}\text{W}\text{h}-1.579\:\text{M}\text{W}\text{h}=0.901\:\text{M}\text{W}\text{h}/\text{d}\text{a}\text{y}$$

5 $$\:{\text{E}}_{\text{s}\text{a}\text{v}\text{i}\text{n}\text{g}}\text{\%}=\frac{0.901}{2.480}=36\:\%$$

### The actual measurements using the power analyser at the plant

The power analyzer is connected to the input of motor (two) for 24 h to measure the total energy consumption by the motor. The power analyzer is connected two times, once while controlling with throttling for 24 h and once while controlling with VSD for 24. The energy consumptions with throttling and VSD are compared to calculate the energy saving.

### Measurements with throttling

Table 6 shows the power analyzer’s measurements of voltage, power, and cumulative power consumed daily at different times of the day when using the throttle flow control method. The result is that the power consumed during the day is constant despite the choke of water flow by the throttling valve, and thus the total power consumed is very high, reaching 2466 KWH during the day.

**Table 6:** Power analyzer measurements while using the flow throttle.

Measurement time	10:01:20			24:04:06		
Line to line voltage (rms)	L_12_	L_23_	L_31_	L_12_	L_23_	L_31_
The value of the line to line voltage	409	413	416	409	409	411
phase voltage (rms)	L_1_	L_2_	L_3_	L_1_	L_2_	L_3_
Value of the phase voltage (Vrms)	169	173	190	169	165	174
Power factor	0.87			0.87		
Frequency (Hz)	50.149			49.98		
Total power consumed over 12 h (kω)	110.9			103		
Total energy consumed over 12 h (kωh)	1561			–		
Total energy consumed over 24 h (kωh)	–			24,666		

### Measurements with VSD

A VSD system is installed in the water station to control the speed of the second motor and then control the flow of the second pump to match the network’s water demand. The power analyzer measures voltage, power, and cumulative power at different times of the day, as shown in Table 7. From the table, using VSD to control the motor speed with a v/f constant makes the power consumed during the day low for the same consumer, and thus the power consumed during the day is also low, reaching 1521 KWH.

**Table 7:** Power analyzer measurements with VSD.

Measurement time	10:01:20			24:04:06		
Line to line voltage (rms)	L_12_	L_23_	L_31_	L_12_	L_23_	L_31_
The value of the line to line voltage	376	376	376	409	409	411
phase voltage (rms)	L_1_	L_2_	L_3_	L_1_	L_2_	L_3_
Value of the phase voltage (Vrms)	122	122	122	123	120	123
Power factor	0.73			0.77		
Frequency (Hz)	40.68			46.59		
V/F constant	376/40.68 = 9			411/46.5 = 8.8		
Total power output over 12 h (kω)	55.2			67.3		
Total energy output over 12 h (kω h)	679.9			1521		

The measured energy consumption per day from power analyzer after installing the VSD as a speed controller is 1521 kWh.

Energy saving calculations upon power analyzer measurements, Ref.^13^.

6 $$\:{\text{E}}_{\text{s}\text{a}\text{v}\text{i}\text{n}\text{g}}\left(\text{k}\text{W}\text{h}\right)={\text{E}}_{\text{b}\text{e}\text{f}\text{o}\text{r}\text{e}\:\text{V}\text{S}\text{D}}\left(\text{k}\text{W}\text{h}\right)-\:{\text{E}}_{\text{w}\text{i}\text{t}\text{h}\:\text{V}\text{S}\text{D}}\left(\text{k}\text{W}\text{h}\right)$$

7 $$\:{\text{E}}_{\text{s}\text{a}\text{v}\text{i}\text{n}\text{g}}=2466-1521=945kWh/DAY$$

8 $$\:{\text{E}}_{\text{s}\text{a}\text{v}\text{i}\text{n}\text{g}}\text{\%}=\frac{{\text{E}}_{\text{s}\text{a}\text{v}\text{i}\text{n}\text{g}}\left(\text{k}\text{W}\text{h}\right)}{{\text{E}}_{\text{b}\text{e}\text{f}\text{o}\text{r}\text{e}\:\text{V}\text{S}\text{D}}\left(\text{k}\text{W}\text{h}\right)}=\frac{945}{2466}=38.32\%$$

The actual measurements and the results of the MATLAB/SIMULINK have some variations and it may be referring to:

- The loading conditions are taken by the average values not by the actual loading values.
- The inverter type in the plant is two levels inverter since the simulation inverter is five levels CHB.

## Conclusion

In this work, energy saving was achieved for a drinking water purification plant in a village in southern Egypt. One of the water pumps was controlled once by the traditional method and once by VSD technology, and after comparison, the results were as follows:

- A significant energy saving of approximately 30% is achieved by installing a VSD according to the affinity law.
- (N_new_/N_old_)^3^ = Kω_new_/ Kω_old_, which means that a small decrease in speed results in a significant decrease. In energy (energy saving).
-The starting torque fluctuates in the traditional way, from 200 to -200 Nm, while the VSD technology has, the starting torque fluctuates between 100 to − 100 Nm.
- The starting current in the traditional method is very high, as in Fig. 7a and b. However, with VSD technology, it fluctuates in the range of 300 amps, and during normal operation, it ranges from 250 to 150 A, depending on the load as in Fig. 13a,b.

## Data Availability

The datasets used and/or analyzed during the current study are available from the corresponding author on reasonable request.
